# Data dictionary services in XNAT and the Human Connectome Project

**DOI:** 10.3389/fninf.2014.00065

**Published:** 2014-07-03

**Authors:** Rick Herrick, Michael McKay, Timothy Olsen, William Horton, Mark Florida, Charles J. Moore, Daniel S. Marcus

**Affiliations:** Neuroinformatics Research Group, Department of Radiology, Washington University School of MedicineSt. Louis, MO, USA

**Keywords:** XNAT, ontologies, translations, publishing, human connectome, human computer interaction

## Abstract

The XNAT informatics platform is an open source data management tool used by biomedical imaging researchers around the world. An important feature of XNAT is its highly extensible architecture: users of XNAT can add new data types to the system to capture the imaging and phenotypic data generated in their studies. Until recently, XNAT has had limited capacity to broadcast the meaning of these data extensions to users, other XNAT installations, and other software. We have implemented a data dictionary service for XNAT, which is currently being used on ConnectomeDB, the Human Connectome Project (HCP) public data sharing website. The data dictionary service provides a framework to define key relationships between data elements and structures across the XNAT installation. This includes not just core data representing medical imaging data or subject or patient evaluations, but also taxonomical structures, security relationships, subject groups, and research protocols. The data dictionary allows users to define metadata for data structures and their properties, such as value types (e.g., textual, integers, floats) and valid value templates, ranges, or field lists. The service provides compatibility and integration with other research data management services by enabling easy migration of XNAT data to standards-based formats such as the Resource Description Framework (RDF), JavaScript Object Notation (JSON), and Extensible Markup Language (XML). It also facilitates the conversion of XNAT's native data schema into standard neuroimaging vocabularies and structures.

## Introduction

XNAT provides a robust and advanced set of tools for searching and filtering the data that it manages (Marcus et al., 2007)^1^. This includes searching for a project or a set of projects, for subjects that meet particular criteria, and for imaging sessions or subject assessments that match a complex set of attributes. Users can join searches across system object types to search on combinations of properties including subject attributes, imaging modality, and assessed demographic or clinical conditions.

This search function was originally created to work with the attributes and properties of the core system data types. This works well enough for a standard XNAT installation, but users often need to add custom field definitions to existing data types and custom data types to represent domain- or project-specific imaging modalities and patient or subject assessments. Further, XNAT's data types and objects are maintained internally in a verbose and fairly complex Extensible Markup Language (XML)-based^2^ structure.

In addition, the advanced search functions in XNAT are quite technical in their presentation and workflow design, so users need a detailed understanding of the underlying data. This search interface works well for experienced XNAT users, but, it is a significant barrier to users who are knowledgeable about the research domain but unfamiliar with XNAT in general or with the specific data models for a particular project.

This became a critical issue for the Human Connectome Project (HCP) (Van Essen et al., 2012)^3^. The HCP public data distribution site is ConnectomeDB^4^, an XNAT instance that delivers curated content to users and leverages the underlying XNAT data structures to define groups of subjects within the overall research population, such as a group of 40 unrelated subjects and a group of 120 (some related) individuals, as well as data sets of particular interest, such as group average resting state connectivity, task fMRI data, and behavioral data scores (Marcus et al., 2013).

The target audience for ConnectomeDB is assumed to be sophisticated in its understanding of the data produced by HCP data acquisition and processing, but, unlike experienced XNAT users, cannot be assumed to have anything other than a basic level of skill with XNAT's search and retrieval features and little or no familiarity with the general XNAT and HCP-specific data models.

This issue—translating concepts, terminology, and operational paradigms specific to XNAT into vocabularies appropriate to other contexts—is one that the XNAT team has encountered frequently as the field of electronic medical imaging has grown and research organizations have become more connected. This has led to greater interest in being able to access and reference data from researchers around the world, without the archive and data management software imposing lexical or syntactical barriers to translation. This is the motivation behind INCF efforts to promote metadata and standards for data sharing within the medical imaging research community (Poline et al., 2012). We took the opportunity afforded by our immediate problem to solve the larger problem in front of us: to transform XNAT from a relatively isolated data archiving repository into an adaptable, query-able, and standards-based data sharing, aggregation, and distribution service.

## Development goals

One of the key features of ConnectomeDB is the subject dashboard. The dashboard provides an intuitive way to search for subjects based on demographics, clinical assessments, and other relevant data, with textual cues and cumulative specification of search criteria. In a standard XNAT installation, users define custom subject groups by defining reusable queries using the advanced search feature. The requirements for publishing to a more generalized audience required a more textual and intuitive means of achieving the same goal.

**Figure 1:**
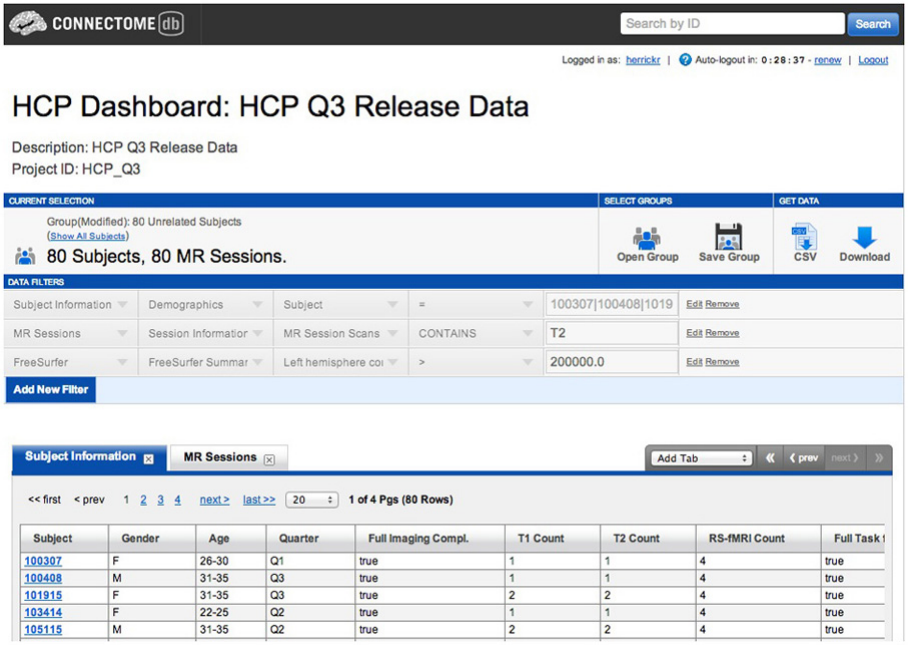
**HCP subject dashboard**.

The subject dashboard lets users create one or more data filters to identify subject groups of interest (Figure 1). Each filter works on some instrument associated with the subjects in the system. These instruments can be directly related to the subject, such as demographics or other subject metadata; extrapolated from subject performance or evaluation, such as performance on cognitive awareness tests, personality evaluations, or physical exams and evaluations; or extracted from imaging data associated with the subject, including types of acquired imaging data, attributes of the data, and processed and secondary capture data.

We needed to make this sort of rich search functionality available to a general audience in a readily understandable way. The *IBM Dictionary of Computing* defines a data dictionary as a “repository of information about data such as meaning, relationships to other data, origin, usage, and format^5^.” In this reading, a data dictionary provides a layer of meaning and context above simple presentation of the available data types and attributes in the system. This enables the association of natural-language synonyms, descriptions, and relational definitions onto the data attributes and structures in the XNAT data store. The initial development goal thus became creating a framework that could enable the definition of the entities within a specific dictionary instance, but remain general in its design, allowing the framework to be re-purposed for other applications leveraging the advantages of a well-defined contextual and descriptive structure for their particular object structure, attributes, and relationships.

In the course of ConnectomeDB development, a second distinct but related application of data dictionary functionality arose. The subject dashboard allows users to define groups of subjects by various attributes of the subjects. Although measures have been taken to protect the identities of the subjects in the HCP study—anonymized subject identifiers, five-year age bands, and other abstractions from direct personal characteristics—the richness of the available demographic and physical data on each subject raises the possibility that users could piece together enough details about subjects that the data could be considered individually identifiable.

The administrative solution for this was to limit the types of subject data available to standard ConnectomeDB users. Although all users of the site must consent to a data-use agreement to access even the “open access” data, users who require access to data deemed to be especially sensitive must accept an additional data-use agreement with more stringent terms.

Accepting and recording acceptance of restricted data-use agreements is just the first step. To implement tiered access to subject data, the system needs to define what those tiers are, as well as what types of subject attributes, assessment instruments, imaging data, etc., are available to each tier. The data dictionary provides a convenient means of defining the tier restriction of entities within the system. The scope and comprehensiveness of the system object hierarchy allows for a great deal of flexibility in how data access may be restricted by tier. This means that ConnectomeDB can use a broad brush to shield entire categories of objects from access, but in other cases can limit access to only a single attribute on a particular item. The data tiers currently defined for ConnectomeDB are:

- Open access.
- Restricted access, for attributes that contain data that could be potentially identifying.
- Sensitive access, which includes data like drug screening and family history of mental illness.
- Confidential access, which is inappropriate for any release, such as record of criminal behavior.

**Figure 2:**
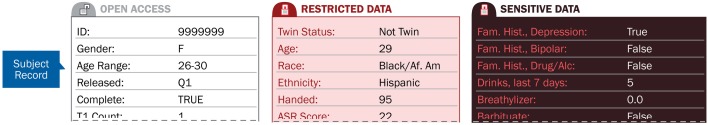
**Access tiers secure subject attribute data based on the level of user data-use agreements**.

Figure 2 shows how the attributes of a resource, such as data collected on a particular subject, are categorized into access tiers. These tiers can then be used to allow access to particular types of information only to users approved to view the data in that tier.

## Implementation

### Frameworks and platforms

Creating an XNAT-specific framework allowed us to complete development and testing of the new service within the tight development timeframe, while also achieving the goals of fitting within our existing development technologies and semantic context, and maintaining our ability to continue to release XNAT with minimal external dependencies.

**Figure 3:**
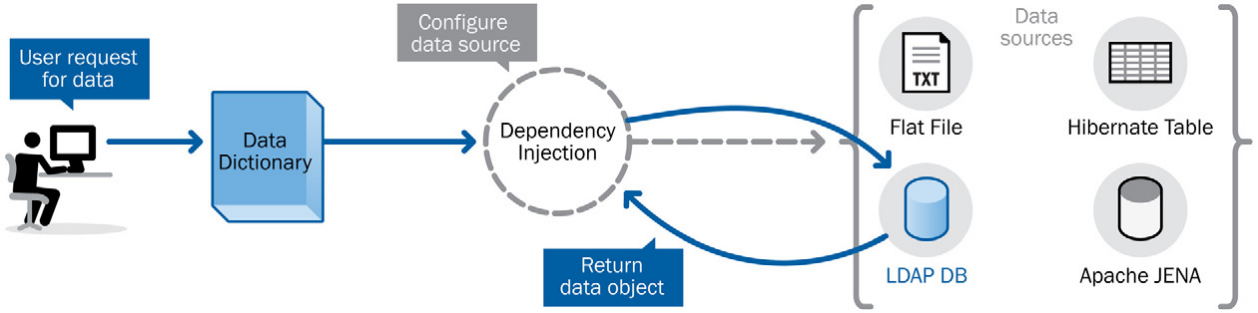
**Dependency injection allows service access while providing flexibility in implementation**.

The XNAT data dictionary was written as an abstract service definition, specifying the contract between the service and its clients while still allowing for flexibility in the implementation. Recent development work on the core XNAT platform has relied primarily on a configuration framework that enables switching between different implementations of a service definition based on the requirements and resources available to a particular deployment—a concept known as *dependency injection*. Figure 3 shows how the data dictionary can be accessed via a conceptual interface that abstracts the functionality, while the actual core back-end functionality can be implemented in a number of different ways.

The contract for the XNAT data dictionary service is defined through a few simple interfaces and entity definitions, while the initial implementation of that service is defined in a concrete implementation class. This leaves the path open to creating future implementations that do utilize the power of stand-alone triplestore servers like Apache Jena, but with the advantage of an API that fits more easily into the context of the XNAT development framework.

### Details

The data dictionary service is implemented as a Java library, with an abstract interface that defines the basic operations of the service. These service operations are performed on a small set of entity types, defined as Java beans, simple data objects that amount to a list of object properties along with functions to retrieve the value of those properties and, in many cases, set the value for those properties. These Java beans are then made available in XNAT through direct calls to the library from within XNAT and through an extension of the XNAT web services API for access from the client-side user interface. A specific instance of a data dictionary is defined in a JavaScript Object Notation (JSON)-formatted^6^ configuration file that is read by the dictionary service on initialization.

### Nodes

The basic building block of the dictionary elements is the node entity. Nodes include a number of core properties that are used by all objects in the dictionary service, shown in Table 1.

**Table 1:** **Node attributes**.

**Property**	**Description**
Name	The name of the node.
Description	A description of the contents of the node.
Position	The suggested position of the node relative to its siblings.
Column header	A shortened version of the node name for display in column headers and restricted display areas.
Projects	A list of XNAT project identifiers with which the node is associated. Not all entities within the data dictionary may be applicable to all projects. For example, as new data models are added to data sets, particular nodes may only be applicable to the later releases that include those models.
Tier	Indicates the security access level required to view or query on the node.

The service includes three specific node types: categories, assessments, and attributes. These nodes are organized in a simple hierarchy and contain additional hierarchy-level specific properties.

A category maps to a research domain and contains a group of assessments. This is the highest level of organization and so encompasses the largest conceptual groupings in the data dictionary.

An assessment node defines a set of related observations about a subject. Assessments are any discreet collection of data that evaluates or describes some aspect of the assessed subject. An assessment defined in the data dictionary generally maps to a standard XNAT or HCP-specific data object, such as subject demographics, clinical assessment, imaging data, and so on. However, mapping is not defined at the assessment level but at the attribute level, so it would be possible to create a data dictionary assessment that actually comprises multiple data types. For example, a clinical assessment type might combine an attribute from direct clinical observations such as MMSE or an IQ assessment with attributes from a genetic or demographic assessment.

An assessment is normally defined within XNAT by its relationship to its project and subject, but the data dictionary service adds an extra definition for the assessment's category. This allows the data dictionary service to group similar assessments for conceptual and organizational purposes. This relationship to a category is the primary and only metadata contained in the assessment dictionary type outside of the base node attributes.

An attribute node defines a particular evaluation or observation. Attributes are the various data points and measurements that make up the content of an assessment. Each attribute in the data dictionary maps directly to a field contained in a core XNAT or HCP-specific data type. Attribute definitions also provide a number of ways to help users enter valid values for the attribute. For example, a list of valid comparison operators restricts the types of operations users can perform against the value of the attribute, a list of valid attribute values limits the values that can be set for the attribute, and so on. The combination of these various types of user assistance provides unobtrusive guidance and assistance to users when navigating the search function on the subject dashboard. **Table 4** shows the full list of properties on the data dictionary attribute definition.

**Figure 4:**
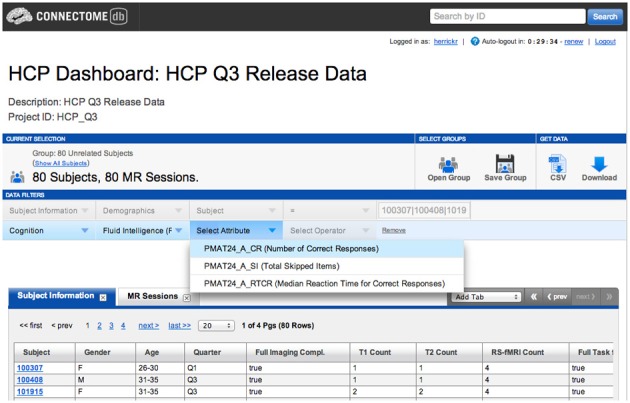
**Category, assessment, and attributes are browse-able through the ConnectomeDB dashboard**.

An example of a full relationship would be the category of Cognition, which includes a number of assessments, such as Fluid Intelligence, which in turn includes a number of attributes, including number of correct responses, total skipped items, and median reaction time for correct responses. The representation of this relationship is illustrated in Figure 4.

Each level of data dictionary entity can be defined with a set of attributes recognized by the data dictionary service. Tables 2–4 describe the unique properties available on each specific node implementation.

**Table 2:**
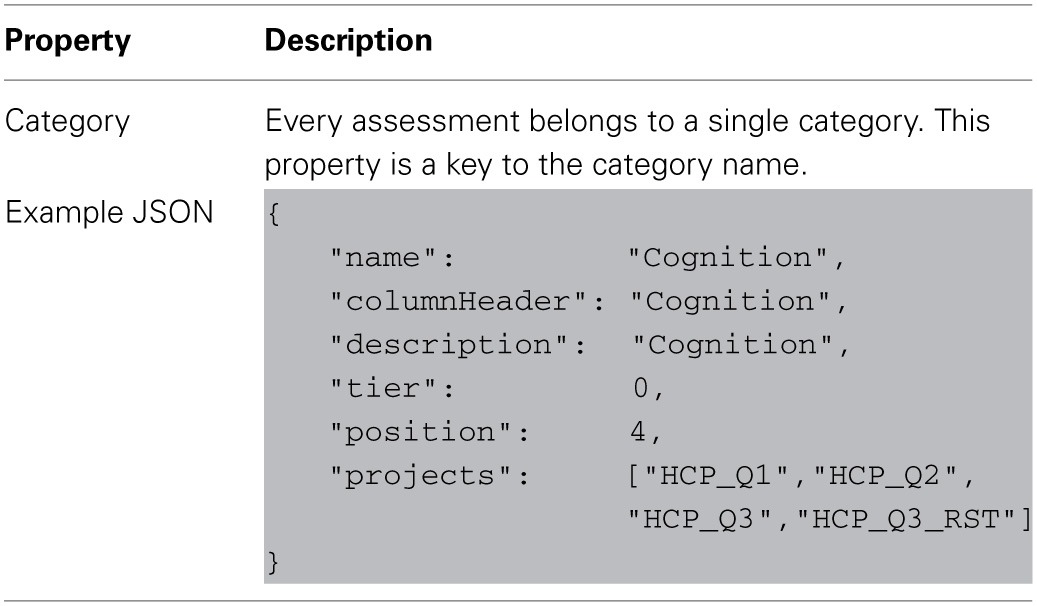
**Defining category node-type properties in data-dictionary**.

**Table 3:**
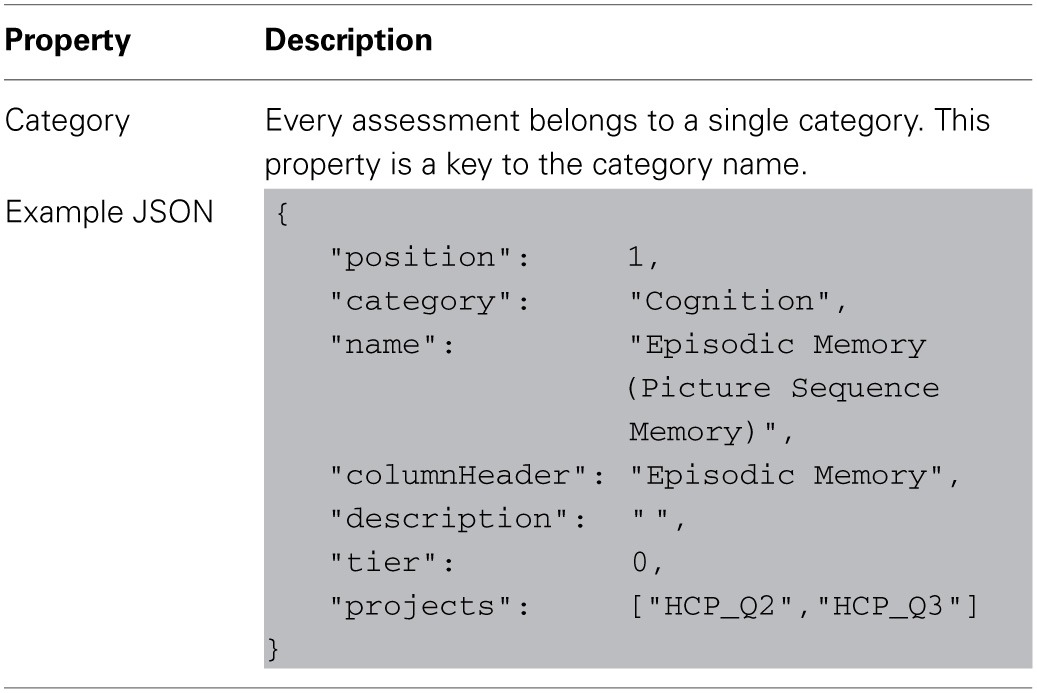
**Defining assessment node-type properties in data-dictionary**.

**Table 4:**
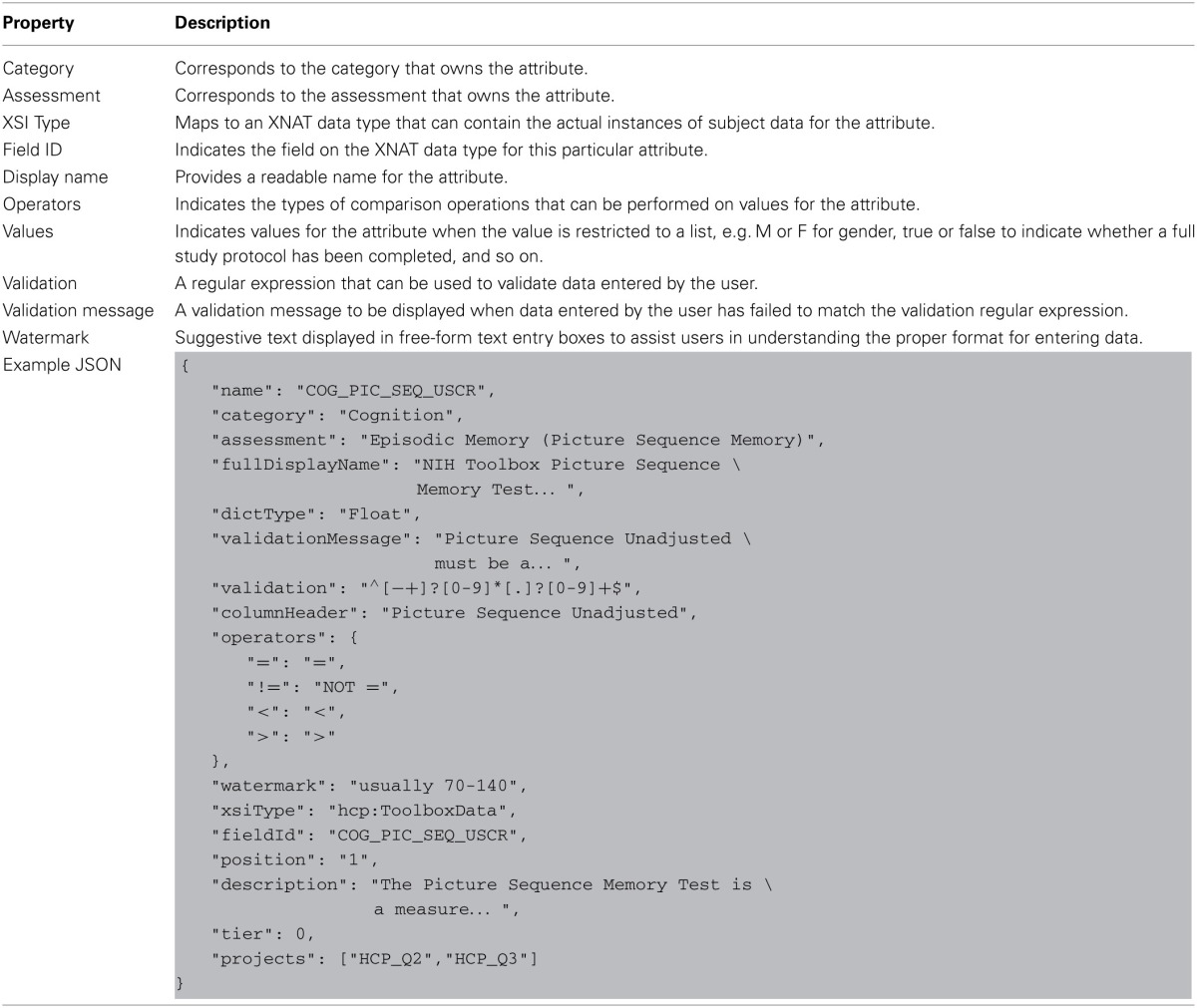
**Defining attribute node-type properties in data-dictionary**.

This relatively simple hierarchical structure provides a framework for defining, navigating, and, most importantly, searching and querying all of the data in ConnectomeDB in a very user-friendly manner. The specific instances of categories, assessments, and attributes map very closely to the complex data type definitions in XNAT that the advanced search functions are designed for, but the descriptive and contextual metadata defined in the data dictionary makes the search functionality closer to natural language.

Once we have defined the data dictionary entries, the configuration is deployed to the server. At that point, the categories, assessments, and attributes are available through the XNAT data dictionary service and can be accessed by any other service that wants to use them. The means by which client services access the data dictionary depends on the relationship to the XNAT server.

For internal XNAT services, that is, services that execute on the same application server and within the same process space as the data dictionary service, there is a programmatic service that can be accessed through XNAT's standard application context. This allows querying of the various entities within the data dictionary, translation of data dictionary entities into addressable XNAT data types and attributes, and rendering of the data dictionary into various data interchange formats (currently the data dictionary service supports only JSON as an interchange format, but future development efforts will extend the available formats to allow for integration with non-XNAT systems and querying tools).

The data dictionary service also provides a Web service that allows services and tools outside of XNAT full access to the metadata and structures in the data dictionary. This provides a means to explore the structures that are defined in the data dictionary. For example, the search filter function shown in Figure 3 is essentially a means of browsing through the data dictionary entities representing the categories of assessments and attributes. It also functions as a translational layer from a conceptual entity—such as a particular attribute and potential values for that attribute—to an actionable data object within the XNAT system. In this view, the XNAT data dictionary service works as a translational tool: rendering technical or domain-specific terminology and nomenclature into formats or language more suited to a particular audience or type of user.

### Application

The first and most obvious usage of the data dictionary's web services API is in the various user interface elements on ConnectomeDB. The search filters in Figure 3 act as a browser for the various data dictionary entities. Once a user has composed a search operation of one or more filters, the query parameters may be checked against validation expressions associated with the attributes. And once the query has been successfully validated, the data dictionary translates the specified data dictionary attributes into XNAT-specific search queries that be run against the server's data store. This makes it much easier for researchers to work with language and concepts with which they are well acquainted to leverage the functionality of XNAT's search and data retrieval services.

**Figure 5:**
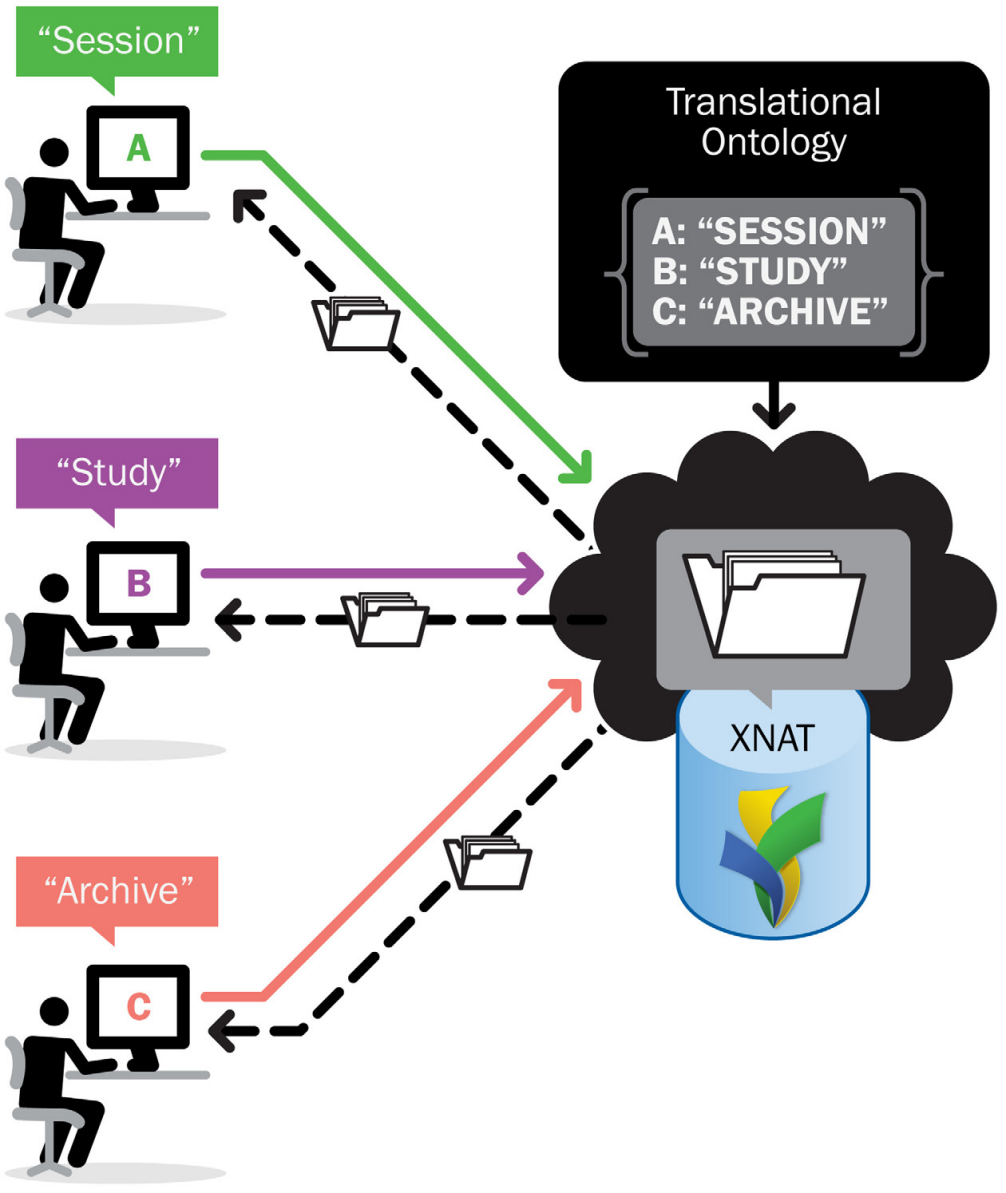
**Translation of different vocabularies to XNAT entities**.

But one of the XNAT development team's far-reaching goals is seamless integration with medical imaging and electronic data capture systems across research organizations. The XNAT data dictionary REST service can help achieve this goal precisely through the same translational function that allows for greater ease of use for human users. Many applications have differing terminology and particular means of structuring, storing, and retrieving data and metadata. There are groups working to standardize the terminology and ensure interoperability amongst those applications, since the end goal for research data services is almost always to make the data as available as possible to the greater research community. It is at that point that these differences in structure and verbiage need to be negotiated and bridged. The XNAT data dictionary services provide a flexible means of mapping these other vocabularies and structures onto XNAT's internal structure, as shown in Figure 5.

## Deployment

The XNAT data dictionary service was initially deployed with the HCP Q3 data release^7^. The service as deployed on ConnectomeDB comprises the following components:

- The core data dictionary service definition and implementation, which provides the underlying metadata persistence and retrieval service.
- The data dictionary REST API, which provides access to the data dictionary service via HTTP.
- A data dictionary search service that returns XML used to build and decorate the tables containing the results of searches based on criteria defined in the data dictionary.

The code and configuration files for these can be found in the Mercurial repository for ConnectomeDB customizations at https://bitbucket.org/hcp/db_builder_customizations. The following sections reference particular code components and configurations from this repository.

### Core data dictionary service

The core data dictionary service is defined by the **DataDictionaryService** interface and implemented for this deployment in the **SimpleDataDictionaryService** class. Upon instantiation, the **SimpleDataDictionaryService** loads a statically defined JSON configuration, contained in the **datadictionary-context.xml** configuration file, to construct and manage the system-wide data dictionary.

This simple service has the advantage of being portable and lightweight, requiring no database connection, persistence layer, or transaction management. Its disadvantage is a lack of flexibility and difficulties in maintaining and extending the data dictionary. Given the specific nature of the HCP deployment and the project's well defined study protocol and set of data types, we opted for the simplest implementation at the cost of extensibility. However, as described earlier, the abstraction of the service interface and configurability of service libraries through dependency injection allows for relatively easy switching between different implementations. This will ease the migration of the service to the XNAT platform, which requires more configurability and extensibility than a statically defined library offers.

The core data dictionary service is accessible through direct calls to its API. The classes as currently constituted aren't available as a stand-alone library.

### REST API

The REST API is the primary means by which clients of the data dictionary access the service. In the case of ConnectomeDB, service clients consist almost exclusively of authenticated users accessing the data dictionary through their browser as part of a login session on the Web site, but, unlike actual data from the HCP study, the data dictionary service can be accessed without authentication by calling the appropriate URLs directly.

The REST API can be accessed through a number of different URIs into the system (in the following table, all calls are relative to the root of the Web service, which in the case of ConnectomeDB is https://db.humanconnectome.org). In the list below, italicized terms are replaced by specific argument values that indicate what specific data the REST call should return.

#### /data/services/ddict/*tier*

This function returns a list of data in the specified tier. The ConnectomeDB data dictionary includes only two separate tiers, **categories**, which returns the top-level categories in the data dictionary, and **attributes**, which returns the whole data dictionary in a single JSON structure. The categories tier can be used to drill down into specific categories all the way down to the attribute level in an efficient way, while the attributes tier is used to retrieve all data in single operation. The first approach is very efficient in terms of the amount of data retrieved on each separate call to the service, while the second approach is efficient in terms of limiting the number of HTTP calls between the server and the REST client.

#### /data/services/ddict/*tier*/*category*

This function gets a list of all metadata subordinate to the indicated category in the tier. In the ConnectomeDB implementation, the only tier for which this call is valid is **categories**, since getting all attributes for all categories is synonymous with retrieving the full **attributes** tier. For example, to find all assessments associated with the FreeSurfer category, the appropriate REST URI would be **/data/services/ddict/categories/FreeSurfer**.

#### /data/services/ddict/*tier*/*category*/*assessment*

This function gets a list of all metadata subordinate to the indicated assessment. For example, to find all attributes associated with the FreeSurfer Volume assessment, the appropriate REST URI would be **/data/services/ddict/attributes/FreeSurfer/Volume**. Note that calling this URI with the **categories** tier effectively ignores any arguments to the right of **category**.

#### /data/services/ddict/*tier*/*category*/*assessment*/*attribute*

This function gets the metadata associated with a particular attribute. This is an efficient way to retrieve the metadata about a specific attribute when you know the category and assessment to which the attribute belongs. For example, to get the metadata about the cerebral spinal fluid volume measure on the FreeSurfer assessment, the appropriate URI path would be **/data/services/ddict/attributes/FreeSurfer/Volume/_CSF**.

#### /data/services/ddict/*tier*/*category*/*assessment*/*attribute*/*validate*/*value*

This REST call provides validation for particular attribute values. Part of the optional metadata that can be associated with an attribute is a regular expression to test a submitted value. If the specified attribute has a validation expression, this method tests the argument for **value** against that regular expression and returns an error if the value doesn't match properly.

### Data dictionary search service

This service provides only a single REST function, **/data/services/search/ddict/*category***. This returns all attributes for the indicated category in XML form. This XML is formatted specifically to be used by the search results table on the subject dashboard and is a good example of how the data dictionary can be used to manage the display of user interface elements.

## Lessons learned and future development

At the start of development of the data dictionary service for the HCP public site, we took the position that our first development efforts would amount to a test run and learning experience to drive future development efforts to create a full-fledged data dictionary and metadata management framework for the XNAT platform. Because release of this framework with ConnectomeDB would not define and restrict future development efforts in core XNAT platform development, we were free to experiment with different approaches to managing the data dictionary, as well as the metadata's relationship to the primary XNAT domain objects such as imaging sessions, research subjects, subject assessors, etc. We also were not restricted by future development goals related to data dictionary implementation, such as supporting export to Resource Description Framework (RDF)^8^, triplestore integration, or import and export operations to and from other medical imaging platforms through mapping and translation of XNAT's internal data structures and taxonomies into common vocabularies and taxonomies.

The primary lesson learned from the data dictionary implementation is the significant limitation in implementing the object structure using Java class definitions. In XNAT, building Java code into a deployable application requires a number of build steps. This overhead made the object structure fairly inflexible, with changes to the structure requiring changes to the underlying code, necessitating a new build and deploy of the server software. The next iteration of the data dictionary service will use flexible definitions for the dictionary node definitions themselves. This configurable definition feature will allow an installed XNAT server quickly define data dictionary entity structures along with specific instances of those structures without requiring redeployment of the server and enable developers and administrators of a system to make their data available to other services.

Another lesson, of a more positive nature, is the value of this sort of rich metadata associated with the data types in the system. This was demonstrated when the need arose for restricting access to particular assessment instruments and attributes on those assessments based on the user security level. Adding the restricted access feature was still a significant effort, but was aided significantly by the application of the data dictionary, which was already carrying metadata about the object hierarchy at precisely the level required to add security scoping to data access.

## Conclusion

In implementing the data dictionary service for ConnectomeDB, we were successful in bridging the gap from XNAT's domain-specific and technical terminology and data structures to the vocabulary and entities that are of real interest to the user who want to perform research rather than learning yet another data management tool. The value and ease of use delivered to the site's users were worth the development resources we committed to the implementation of this feature. We also began the process of presenting XNAT and its data not just as a singular software service, but as a flexible and multi-use data repository. The lessons learned from the process of developing the first version of the XNAT data dictionary service are currently being applied in the planning and development of the next generation of the XNAT imaging platform. Most importantly, we need to simplify the process of defining a data dictionary's structures and mapping to XNAT internal data structures. We also will expand the target dialects for translation, with the initial goal of supporting RDF and export of XNAT metadata to standard triplestore services like Apache Jena. By translating from XNAT-specific data to protocols and formats understood by other applications and services, we aim to make XNAT support industry-standard data analysis and mining tools, reporting and visualization frameworks, and modeling and graphics applications. This will allow greater flexibility for researchers to analyze their research data and generated data resources. It will also let XNAT serve as a back-end service for other publishing platforms and research tools. By providing the functions to work as an end-to-end-lifecycle data management tool, we hope to help the research community achieve its core goal of converting basic science into completed research.

### Conflict of interest statement

The authors declare that the research was conducted in the absence of any commercial or financial relationships that could be construed as a potential conflict of interest.

## Footnotes

^1^XNAT, NRG Lab at the Washington University School of Medicine. http://www.xnat.org.

^2^XML Core Working Group (2008). Extensible Markup Language (XML) 1.0 (Fifth Edition) http://www.w3.org/TR/xml/.

^3^The Human Connectome Project. https://www.humanconnectome.org.

^4^ConnectomeDB. https://db.humanconnectome.org.

^5^Dictionary of IBM and Computing Terminology. http://www-03.ibm.com/ibm/history/documents/pdf/glossary.pdf.

^6^JSON is an open standard format with no canonical definition: http://en.wikipedia.org/wiki/JSON.

^7^HCP Q3 Data Release Reference. https://www.humanconnectome.org/documentation/Q3.

^8^RDF Working Group (2014). RDF Schema 1.1 http://www.w3.org/TR/rdf-schema/.
